# Long-term phlebotomy successfully alleviated hepatic iron accumulation in a ferroportin disease patient with a mutation in *SLC40A1*: a case report

**DOI:** 10.1186/s12876-021-01674-z

**Published:** 2021-03-05

**Authors:** Sohji Nishina, Yasuyuki Tomiyama, Katsuya Ikuta, Yasuaki Tatsumi, Yasumichi Toki, Ayako Kato, Koichi Kato, Naoko Yoshioka, Kyo Sasaki, Yuichi Hara, Keisuke Hino

**Affiliations:** 1 Department of Hepatology and Pancreatology, Kawasaki Medical School, 577 Matsushima, Kurashiki, 701-0192 Japan; 2 Laboratory of Medicine, School of Pharmacy, Aichi-Gakuin University, Nagoya, Japan; 3 Division of Gastroenterology and Hematology/Oncology, Department of Medicine, Asahikawa Medical University, Asahikawa, Japan; 4 Present Address: Japanese Red Cross Hokkaido Blood Center, Sapporo, Japan

**Keywords:** Ferritin, Hepcidin, Hereditary hemochromatosis, Nonclassical ferroportin disease

## Abstract

**Background:**

Hereditary hemochromatosis is a heterogenous group of inherited iron-overload conditions that is characterized by increased intestinal absorption and deposition in vital organs. Hepcidin is a soluble regulator that acts to attenuate both intestinal iron absorption and iron release from reticuloendothelial macrophages through internalization of ferroportin-1, an iron exporter. Ferroportin disease is hereditary hemochromatosis which is affected by *SLC40A1*, a gene coding ferroportin-1, and phenotypically classified into two forms (classical and nonclassical). In nonclassical form, ferroportin mutations are responsible for a gain of function with full iron export capability but insensitivity to downregulation by hepcidin. Here, we report a case of nonclassical ferroportin disease.

**Case presentation:**

A 46-year-old Japanese man showed elevated serum iron (284 μg/dl), ferritin (1722 ng/ml), transferrin saturation ratio (91.3%), and hepcidin-25 level (139.6 ng/ml). Magnetic resonance imaging (MRI) demonstrated a marked reduction in the signal intensity of the liver in T1- and T2-weighted images. The liver histology exhibited a large amount of iron that had accumulated predominantly in hepatocytes. We identified a heterozygous 1520A > G (p.H507R) mutation in the *SLC40A1* gene. Phlebotomy (400 ml at a time) was monthly performed for 3 years in this patient. Importantly, the serum hepcidin level (1.0 ng/ml) was normal when the serum ferritin level was normal and hepatic iron accumulation was remarkably reduced after 3 years of phlebotomy.

**Conclusions:**

The present case demonstrated for the first time that there was a correlation between hepatic iron levels as measured by MRI and serum hepcidin levels through long-term phlebotomy in a patient with ferroportin disease with the p.H507R mutation of in *SLC40A1*.

## Background

Hereditary hemochromatosis is a heterogeneous group of inherited iron overload conditions that is characterized by increased intestinal absorption and deposition in vital organs. Hereditary hemochromatosis has been clinically classified into two phenotypes. The classical form induces mainly cirrhosis, diabetes mellitus (DM), and/or skin pigmentation in middle-aged patients, while the other form, juvenile hemochromatosis, results in cardiac failure and hypogonadism before patients reach the age of 30 [1]. On the other hand, four types (types 1, 2, 3, and 4) of hemochromatosis have been genetically classified on the basis of mutations in five genes (*HFE*, *human antimicrobial peptide* [*HAMP*], *hemojuvelin* [*HJV*], *transferrin receptor 2* [*TFR2*], and *SLC40A1*). The molecular mechanism common to all types of hereditary hemochromatosis, except type 4, fails to regulate hepcidin expression in response to cellular iron levels [2]. Hepcidin is a 25 amino-acid peptide hormone exclusively synthesized in the liver and a soluble regulator that acts to attenuate both intestinal iron absorption and iron release from reticuloendothelial macrophages [3, 4]. Hepcidin acts by triggering internalization of ferroportin-1, an iron exporter, which results in its degradation, and the trapping iron in absorptive enterocytes, macrophages, and hepatocytes [5].

Type 4 hemochromatosis, which is affected by mutations in *SLC40A1*, a gene coding ferroportin-1, mutations, is known as ferroportin disease [6]. The inheritance pattern is autosomal dominant. This disease is phenotypically heterogeneous with two forms (classical and nonclassical). In the classical form, the loss-of-function mutants of ferroportin prevent iron export from cells, resulting in hyperferritinemia, a normal to low transferrin saturation, and iron accumulation predominantly in reticuloendothelial cells [6]. In the nonclassical form, ferroportin mutations are responsible for a gain of function with full iron export capability but insensitivity to downregulation by hepcidin, leading to increased transferrin saturation and iron accumulation in hepatocytes in addition to hyperferritinemia [6, 7].

In this report, we present a Japanese patient with nonclassical ferroportin disease who showed a successful response to long-term phlebotomy based on the remarkable decrease in hepatic iron accumulation.

## Case presentation

A 46-year-old Japanese man was referred to Kawasaki Medical School Hospital for further examination of liver dysfunction. The patient had been diagnosed with DM at approximately 35 years of age and treated with anti-DM drugs for the last 5 years. He also had continuous mild elevation of alanine aminotransferase (ALT) of unknown origin during the last 5 years. When the patient met his younger brother several months before his first visit to our department, he found out that his younger brother had been diagnosed with hemochromatosis in another hospital, and was advised to have the etiology of his liver dysfunction examined.

This patient did not have a history of alcohol abuse, blood transfusion, or medication except for anti-DM drugs. His parents had no history of chronic liver diseases, except for his mother, who has DM. The degree of his ALT elevation did not change significantly after the commencement of treatment with anti-DM drugs. His physical examination did not show skin pigmentation, arrhythmia, or hepatosplenomegaly.

Hematological examination revealed no abnormalities. Biochemical examination demonstrated mild elevation of ALT (43 U/l) and moderate elevation of fasting blood glucose (123 mg/dl) and hemoglobin A1c (6.9%). The patient was negative for hepatitis B surface antigen and anti-hepatitis C virus antibody. Importantly, the serum iron level was moderately elevated (284 μg/dl, reference range 40–188 μg/dl), and the serum ferritin (1722 ng/ml, reference range 10–240 ng/ml) and transferrin saturation ratio (91.3%) were remarkably elevated. Serum copper and ceruloplasmin levels were within the normal range. The serum hepcidin-25 level, which was measured using liquid chromatography coupled with tandem mass spectrometry, was remarkably increased (139.6 ng/ml, reference range 7.8 ± 7.0 ng/ml).

**Fig. 1:**
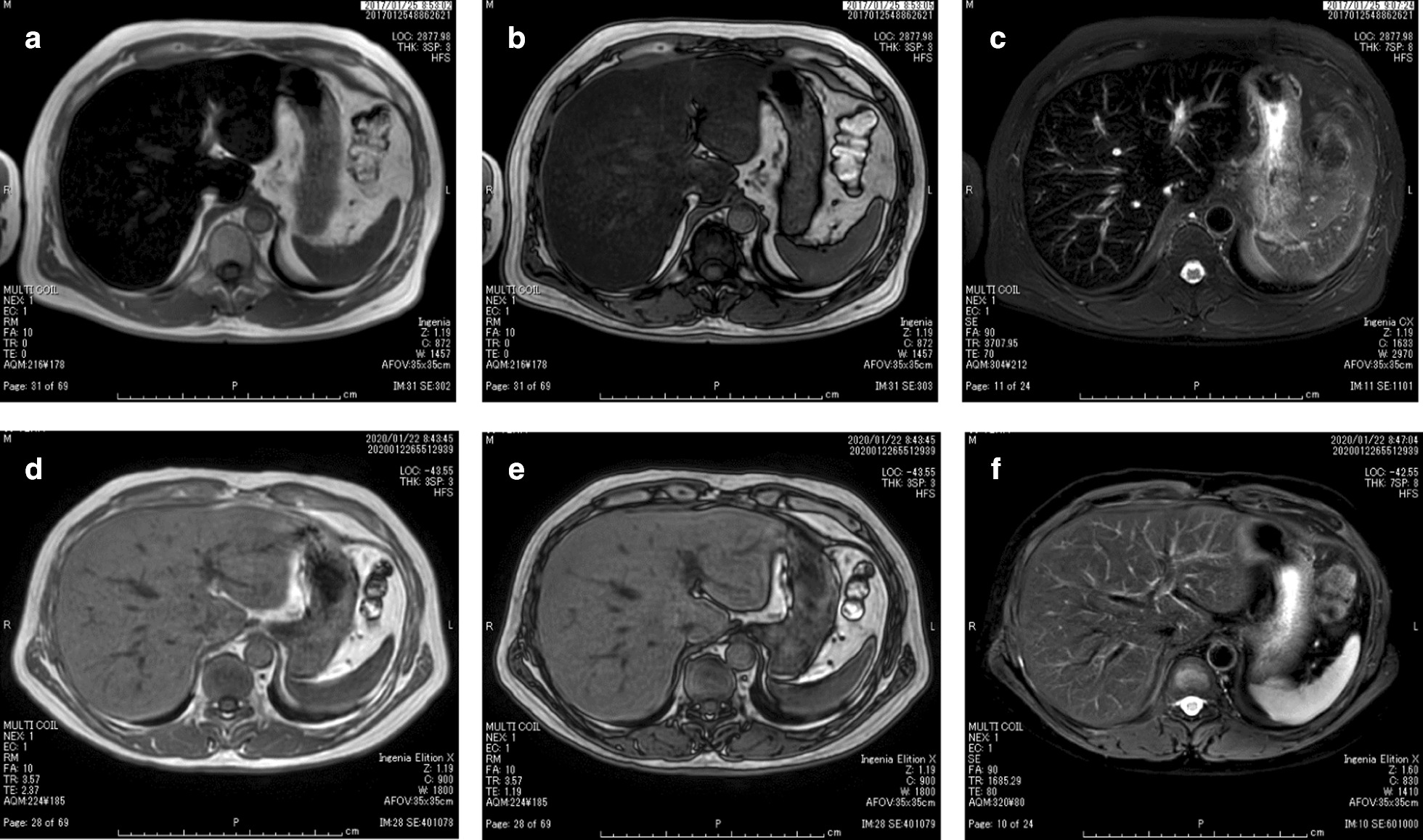
Magnetic resonance imaging of the liver before (2017/01) and after (2020/01) phlebotomy. **a** T1-weighted in-phase image before phlebotomy. **b** T1-weighted out-of-phase image before phlebotomy. **c** T2-weighted image before phlebotomy. **d** T1-weighted in-phase image after phlebotomy. **e** T1-weighted out-of-phase image after phlebotomy. **f** T2-weighted image after phlebotomy

**Fig. 2:**
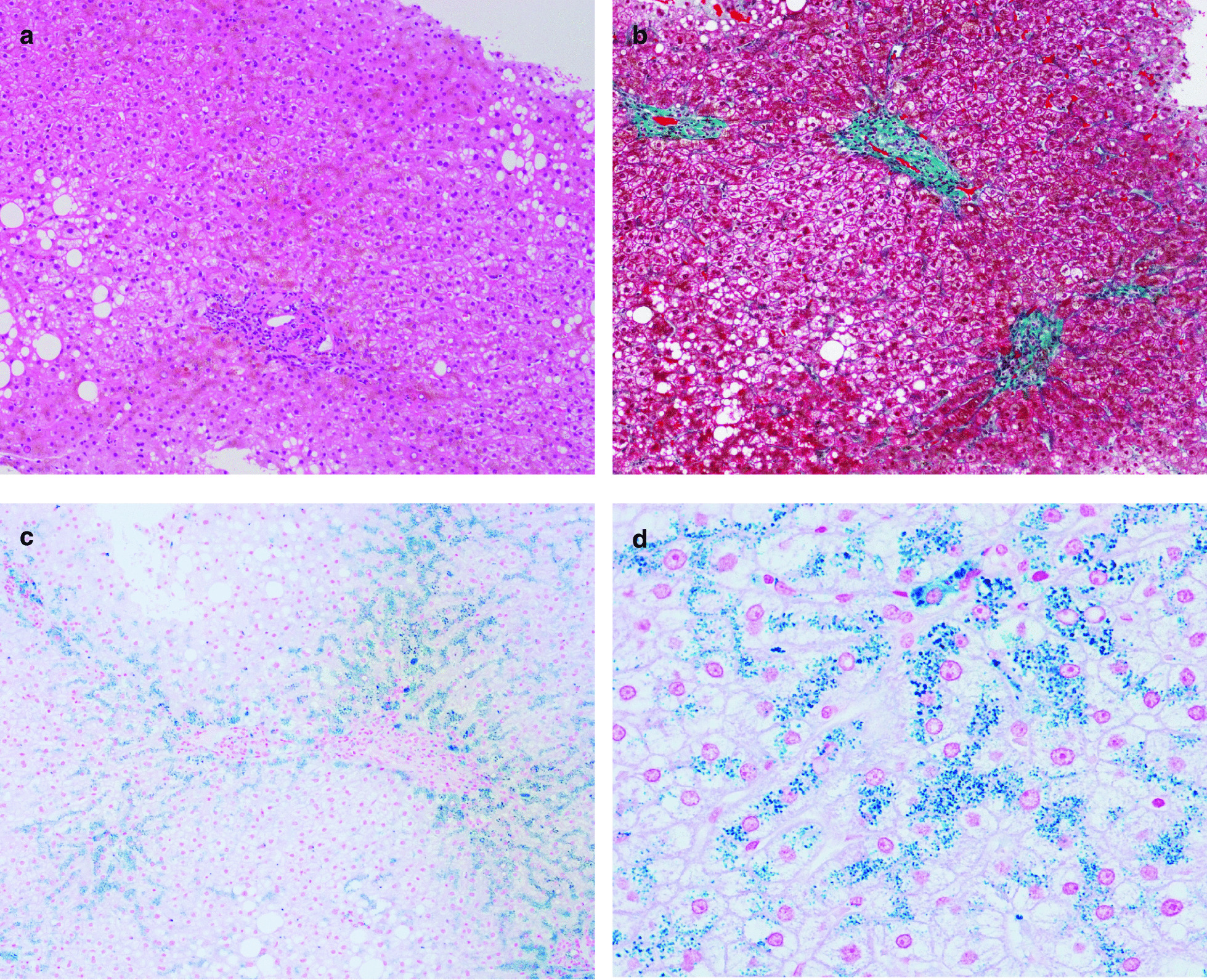
Histology of liver biopsy specimens. **a** H&E staining (× 40). **b** Masson’s trichrome staining (× 40). **c** Prussian blue staining (× 40). **d** Prussian blue staining (× 400)

Ultrasonography demonstrated the mild hepatorenal contrast, suggesting the presence of fatty liver. Magnetic resonance imaging (MRI) showed a marked reduction in the signal intensity of the liver in T1- and T2-weighted images. Furthermore, an in-phase image of the T1-weighted image showed a greater reduction in the signal intensity than an out-of-phase image (Fig. 1a–c), suggesting hepatic iron accumulation, as evidenced in a previous report [8]. Because we suspected that liver dysfunction resulted from hereditary hemochromatosis based on the family history, elevated iron, ferritin and transferrin saturation ratio in serum, and MRI findings, we performed a liver biopsy in this patient after obtaining written consent. The liver histology exhibited mild hepatic steatosis, mild mononuclear cell infiltration (Fig. 2a), and mild to moderate periportal fibrosis (Fig. 2b). Notably, we observed a large amount of iron that had accumulated predominantly in hepatocytes in the liver biopsy specimen (Fig. 2c, d).

**Fig. 3:**
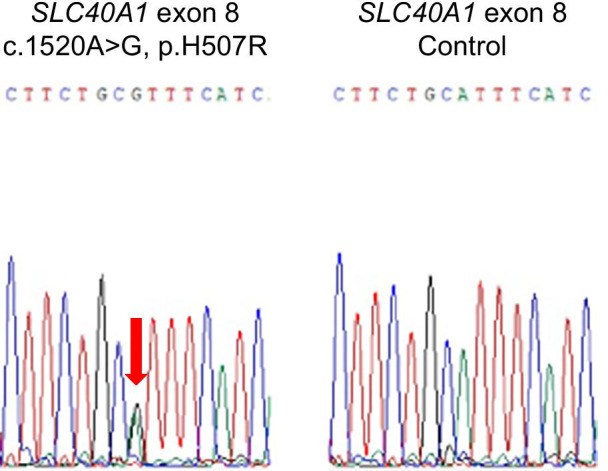
Sequence analysis of *SLC40A1* exon 8 in the patient. This patient had a heterozygous A > G transition at c.1520 in exon 8 (p.H507R) in the *SLC40A1* gene

In hereditary hemochromatosis (except for ferroportin disease), loss of any one of the hepcidin regulator genes (*HFE*, *HAMP*, *HJV*, and *TFR2*) attenuates or abrogates intracellular hepcidin signal transduction and hepcidin secretion [6, 9]. In contrast, hepcidin is appropriately synthesized and released in response to increased serum iron levels in ferroportin disease. We speculated that this patient might have ferroportin disease, since the serum hepcidin level (139.6 ng/ml) was increased in response to elevated serum iron levels. We scanned all the cording regions and the splicing junctions of *SLC40A1*, a gene coding ferroportin-1, after obtaining informed consent and having our genetic examination approved by the Kawasaki Medical School Ethics Committee. This patient had a heterozygous A > G transition at c.1520 in exon 8 (p.H507R) in the *SLC40A1* gene (Fig. 3). Thus, taken together with increased hepcidin-25 level, high transferrin saturation ratio, and iron accumulation predominantly in hepatocytes, we diagnosed this patient with nonclassical ferroportin disease. Unfortunately, we could not examine mutations in *SLC40A1* in his family lineage.

**Fig. 4:**
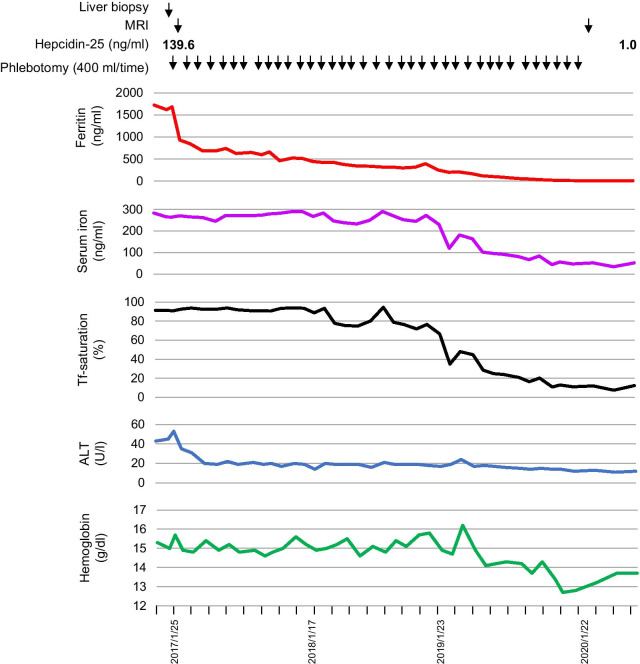
The clinical course of the patient before, during and after phlebotomy, including the change of serum ferritin, serum iron, serum transferrin saturation ratio, serum ALT, and hemoglobin levels

We initiated treatment with phlebotomy in January in 2017. Phlebotomy (400 ml at a time) was performed monthly for 3 years (Fig. 4). The serum ferritin level rapidly declined in a few months and the serum iron and transferrin saturation ratio started to decline in approximately one year after the commencement of phlebotomy, and then gradually decreased until the last phlebotomy. Serum ALT also returned to the normal range in a few months after the commencement of phlebotomy, and remained normal thereafter. The hemoglobin level did not decrease for the first 2 years after the commencement of phlebotomy and then gradually decreased until the last phlebotomy, but slightly increased after the cessation of phlebotomy (Fig. 4). MRI after 3 years of phlebotomy demonstrated a marked restoration in the signal intensity reduction in the liver in T1- and T2-weighted images compared to that before the commencement of phlebotomy (Fig. 1d-f), suggesting a marked reduction in hepatic iron accumulation by phlebotomy. Importantly, the serum hepcidin level (1.0 ng/ml) was normal when the serum ferritin level was normal and hepatic iron accumulation was remarkably reduced after 3 years of phlebotomy (Fig. 4). Serum ALT and ferritin levels have remained normal to date since cessation of phlebotomy (for 6 months).

## Discussion and conclusion

A systemic meta-analysis of ferroportin disease reported that eighty of the 176 individuals with *SLC40A1* mutations were classified as classical phenotype with hyperferritinemia and normal transferrin saturation, and 53 were nonclassical phenotype with hyperferritinemia and elevated transferrin saturation [10]. A patient with mutant ferroportin p.H507R who was originally reported in the United Kingdom showed hyperferritinemia, elevated transferrin saturation and iron accumulation predominantly in hepatocytes (nonclassical form) [11], which is consistent with the present case in this study. Functional analysis of mutant ferroportin p.H507R revealed that p.H507R conferred resistance to hepcidin [11]. Hepcidin binding to ferroportin causes ubiquitination, endocytosis, and degradation of the ligand-receptor complex, thereby decreasing iron supply to plasma [5, 12]. Of note, structure and function analysis of ferroportin demonstrated that nonclassical ferroportin disease was caused by ferroportin mutations that suppress hepcidin binding or hinder conformational changes required for ubiquitination and endocytosis [13]. A mutant ferroportin p.H507R has been shown to have impaired hepcidin-mediated ubiquitination activity, even though it has normal hepcidin binding activity [13]. The association of p.H507R with this phenotype is explained by mapping the residues onto computational models of the human ferroportin structure, which indicated that mutations causing ubiquitination resistance were positioned at helix–helix interfaces, likely preventing the hepcidin-induced conformational change [13]. On the other hand, a recent analysis of cryogenic electron-microscopy indicated a possibility that p.H507R disrupts hepcidin binding to ferroportin [14]. In this report two metal-binding sites within the N and C domains of ferroportin have been identified. The N domain metal site is crucial for iron efflux, whereas the C domain metal-binding site is important for hepcidin binding. The mutation of p.H507R was predicted to disrupt the precise tetrahedral coordination geometry that is required for the metal binding within the C domain [14]. Thus, it seems to be reasonable that the present patient with a mutation of p.H507R in *SLC40A1* exhibited nonclassical ferroportin disease. To the best of our knowledge, based on the literature, the present case is the fifth reported patient with mutant ferroportin p.H507R and the third family lineage in the world [11, 15].

Hepcidin is expressed from the *HAMP* gene located on the long arm of chromosome 19 [9]. The increase in iron levels and inflammation upregulate the transcription of the *HAMP* gene [16–19]. HJV, HFE, TRF1, and TFR2, which are located at the surface of hepatocytes, are considered to be “iron sensors.” The HJV-hepcidin axis is the most important mechanism for the upregulation of *HAMP* expression during iron overload [15]. In this patient, the increased levels of both ferritin and hepcidin-25 in serum suggested that hepcidin secretion was properly regulated, which was consistent with another Japanese patient with mutant ferroportin p.H507R [15]. In addition, we have reported for the first time that serum hepcidin-25 levels can return to the normal range after reduction in hepatic iron accumulation by phlebotomy in this patient.

Phlebotomy is the standard treatment for hereditary hemochromatosis, but there are no evidence-based guidelines on the use of therapeutic phlebotomy. It has been reported that all patients with homozygous hereditary hemochromatosis and evidence of iron overload (i.e., transferrin saturation greater than 45% and serum ferritin level greater than 300 ng/ml in men and greater than 200 ng/ml in women) should be treated, regardless of symptoms [20]. Although this patient had a heterozygous A > G transition at c.1520 in exon 8 (p.H507R) in the *SLC40A1* gene without any symptoms, we treated this patient with phlebotomy since iron overload (91.3% transferrin saturation ratio and 1722 ng/ml serum ferritin) was prominent. Considering that each 500 ml unit of whole blood removes 200 to 250 mg of iron and reduces serum ferritin levels by approximately 30 ng/ml [21], 400 ml of whole blood was removed monthly for 3 years in this patient. The 3-year-phlebotomy successfully alleviated hepatic iron accumulation. We discontinued phlebotomy when the patient had a hemoglobin level of 12.7 g/dl and a serum ferritin level of 16 ng/ml, because phlebotomy is advised to be withheld when the hemoglobin level is less than 12.5 g/dl [16].

In conclusion, we reported a patient with ferroportin disease with the p.H507R mutation of in *SLC40A1* in whom hepatic iron accumulation was successfully alleviated by long-term phlebotomy. The present case demonstrated for the first time that there was a correlation between hepatic iron levels as measured by MRI and serum hepcidin levels through long-term phlebotomy in a patient with ferroportin disease with the p.H507R mutation of in *SLC40A1*.

## Data Availability

All data generated or analyzed for this study are included in this published article.

## Abbreviations

DM: diabetes mellitus
HAMP: human antimicrobial peptide
HJV: hemojuvelin
TFR2: transferrin receptor 2
ALT: alanine aminotransferase
MRI: magnetic resonance imaging
